# Impact of genetic factors on antioxidant rescue of maternal diabetes–associated congenital heart disease

**DOI:** 10.1172/jci.insight.183516

**Published:** 2024-12-06

**Authors:** Talita Z. Choudhury, Sarah C. Greskovich, Holly B. Girard, Anupama S. Rao, Yogesh Budhathoki, Emily M. Cameron, Sara Conroy, Deqiang Li, Ming-Tao Zhao, Vidu Garg

**Affiliations:** 1 Center for Cardiovascular Research, Abigail Wexner Research Institute, and The Heart Center, Nationwide Children’s Hospital, Columbus, Ohio, USA.; 2 Molecular, Cellular and Developmental Biology Program, The Ohio State University, Columbus, Ohio, USA.; 3 Center for Perinatal Research, Abigail Wexner Research Institute, Nationwide Children’s Hospital, Columbus, Ohio, USA.; 4 Department of Pediatrics and; 5 Department of Molecular Genetics, The Ohio State University, Columbus, Ohio, USA.

**Keywords:** Cardiology, Development, Embryonic development, Endothelial cells, Genetic diseases

## Abstract

Congenital heart disease (CHD) affects approximately 1% of live births. Although genetic and environmental etiologic contributors have been identified, the majority of CHD lacks a definitive cause, suggesting the role of gene-environment interactions (GxEs) in disease pathogenesis. Maternal diabetes mellitus (matDM) is among the most prevalent environmental risk factors for CHD. However, there is a substantial knowledge gap in understanding how matDM acts upon susceptible genetic backgrounds to increase disease expressivity. Previously, we reported a GxE between *Notch1* haploinsufficiency and matDM leading to increased CHD penetrance. Here, we demonstrate a cell lineage–specific effect of *Notch1* haploinsufficiency in matDM-exposed embryos, implicating endothelial/endocardial tissues in the developing heart. We report impaired atrioventricular cushion morphogenesis in matDM-exposed *Notch1^+/–^* animals and show a synergistic effect of *NOTCH1* haploinsufficiency and oxidative stress in dysregulation of gene regulatory networks critical for endocardial cushion morphogenesis in vitro. Mitigation of matDM-associated oxidative stress via superoxide dismutase 1 overexpression did not rescue CHD in *Notch1*-haploinsufficient mice compared to wild-type littermates. Our results show the combinatorial interaction of matDM-associated oxidative stress and a genetic predisposition, *Notch1* haploinsufficiency, on cardiac development, supporting a GxE model for CHD etiology and suggesting that antioxidant strategies alone may be ineffective in genetically susceptible individuals.

## Abstract 2

Gene-environment etiology for congenital heart disease is studied. Maternal diabetes and Notch1 haploinsufficiency hinder endocardial cushion development but antioxidants show a differing ability to rescue disease.

## Introduction

Congenital heart disease (CHD) affects approximately 1% of live births and is the leading cause of birth defect–related infant mortality (1). Although high-throughput genome-sequencing technologies have made remarkable advances in uncovering genetic etiologies for CHD, the underlying cause for more than half of CHD cases is still unknown (2–5). A subset of these cases have long been proposed to be the combinatorial effect of genetic predisposition and environmental influences, resulting in complex inheritance patterns and variable CHD expressivity (6, 7). Among environmental contributors, maternal pregestational diabetes mellitus is a highly prevalent and well-established risk factor for CHD, increasing the risk of having an infant with CHD by 3- to 5-fold (8–13). The influence of maternal diabetes mellitus (matDM) on cardiac development has been studied in animal models, shedding light on key cellular and molecular pathways particularly vulnerable to this environmental milieu (14). Recently, matDM has been shown to have cell lineage–specific effects during cardiac morphogenesis, specifically affecting the second heart field and neural crest lineages to disrupt cardiomyocyte differentiation and anterior-posterior specification (15, 16). The incidence of CHD in a pregnancy complicated by pregestational diabetes mellitus has remained elevated even with substantial advancements in prenatal care, suggesting that matDM may cause CHD by acting on susceptible genetic backgrounds, i.e., gene-environment interaction (GxE) etiology. However, the cellular and molecular basis for GxE in CHD as postulated in the multifactorial hypothesis for CHD remain limited (6, 17, 18).

Cardiac development is a dynamic process with tight spatiotemporal regulation of several multipotent cardiac cell lineages (19, 20). Endothelial-mesenchymal transition (EndMT) is a crucial process that occurs early in heart development wherein endocardial cells lining the atrioventricular canal (AVC) and developing outflow tract (OFT) detach from the monolayer and migrate into the preformed cardiac jelly to form endocardial cushions (21–23). Subsequent remodeling of the endocardial cushions at the AVC leads to the formation of the atrioventricular valve leaflets, atrioventricular septum, and membranous portion of the ventricular septum (24, 25). At the OFT, endocardial cushions receive additional contributions from the migrating cardiac neural crest cells to form the semilunar valves and aorticopulmonary septum (26). Although matDM is associated with a spectrum of CHD phenotypes, the most commonly observed include septal and conotruncal (involving OFT and great vessels) heart defects, suggesting EndMT and endocardial cushion morphogenesis are particularly susceptible to disruption by the abnormal diabetic environment (10, 11). This is supported by several published studies in animal models showing dysregulation of key EndMT signaling pathways in embryonic hearts exposed to matDM, including BMP, TGF-β, and Notch signaling (27–31). Additionally, oxidative stress is a hallmark of diabetic embryopathy, and impaired redox signaling has been implicated in dysregulation of several cardiac developmental pathways, suggesting that matDM-associated oxidative stress may be culpable for the elevated risk of CHD (31, 32).

Previously, we found matDM interacts with *Notch1* haploinsufficiency in mice to increase the incidence of membranous ventricular septal defects (VSDs), supporting a GxE between *Notch1* and matDM (27). Pathogenic *NOTCH1* variants are associated with a spectrum of CHD in humans, and Notch signaling is known to interact with other signaling pathways such as BMP and TGF-β to facilitate EndMT in the developing heart (33–38). To better characterize the *Notch1*-matDM GxE and place it in the context of cardiac development, we hypothesized that matDM and *Notch1* haploinsufficiency functionally converge to disrupt endocardial cushion morphogenesis and EndMT to increase the risk of CHD.

In this study, we demonstrate that matDM and *Notch1* haploinsufficiency interact within the developing endothelial/endocardial and endocardially derived mesenchyme to increase the incidence of membranous VSD. We report abnormal atrioventricular (AV) cushion morphogenesis in matDM-exposed *Notch1*-haploinsufficient (*Notch1^+/–^*) embryonic hearts compared with nondiabetic controls. In a human induced pluripotent stem cell–based (iPSC-based) in vitro model, we show that *NOTCH1* haploinsufficiency sensitizes endothelial cells to the effects of oxidative stress and disrupts a network of genes and biological processes underlying EndMT and endocardial cushion morphogenesis. Consistent with this, we find matDM-exposed *Notch1^+/–^* embryos are insensitive to antioxidant-based therapeutic strategy, despite rescue of VSD observed in matDM-exposed WT littermates. Overall, the results from this study elucidate mechanisms by which matDM interacts with a genetic susceptibility, i.e., *Notch1* haploinsufficiency, to bring about cell lineage–specific effects and increase penetrance of CHD. This work serves as experimental proof supporting a multifactorial etiology for CHD, underscoring the need to identify novel genetic modifiers that act in conjunction with environmental teratogens to increase the incidence of CHD, and shows the variability in phenotypic rescue with antioxidant therapies in a genetically susceptible background.

## Results

### GxE between endothelial Notch1 haploinsufficiency and matDM causes CHDs.

Previously, we identified a novel GxE between *Notch1* haploinsufficiency and matDM in mice, wherein *Notch1^+/–^* embryos exposed to matDM had significantly higher incidence of VSD compared with WT littermates at embryonic day (E) 13.5 (27). We first sought to determine if this GxE is evident at a later developmental stage in mice. For this, we generated streptozotocin-induced (STZ-induced) diabetic WT females for timed mating with nondiabetic *Notch1^+/–^* males. Analysis of matDM-exposed embryos at E14.5 (*n* = 3 litters) verified a previously reported GxE resulting in a significantly higher incidence of membranous VSD in *Notch1^+/–^* (10/14, 71%) embryos compared with WT littermates (1/10, 10%, Fisher’s exact test *P* value = 0.005) (Supplemental Figure 1, A–C; supplemental material available online with this article; https://doi.org/10.1172/jci.insight.183516DS1).

**Figure 1:**
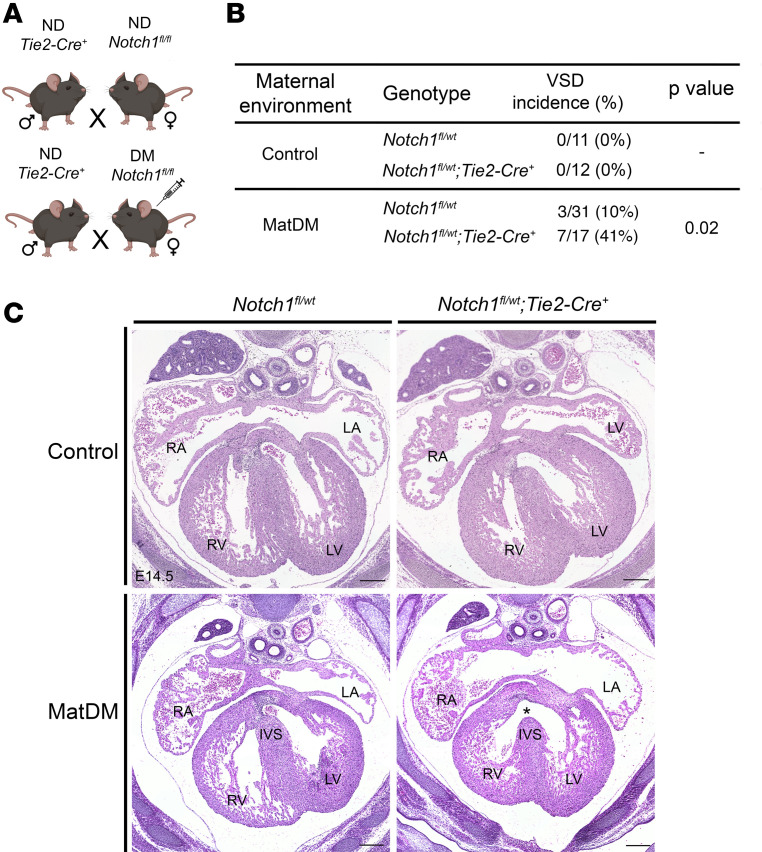
Endothelial/endocardial haploinsufficiency of *Notch1* is sufficient for GxE with matDM. (**A**) Breeding scheme to generate diabetic and nondiabetic E14.5 *Notch1^fl/wt^* and *Notch1^fl/wt^ Tie2-Cre^+^* mice for histological analysis. (**B**) Table showing incidence of VSD in E14.5 embryos. (**C**) Representative images of matDM-exposed and nondiabetic *Notch1^fl/wt^* and *Notch1^fl/wt^ Tie2-Cre^+^* embryonic hearts. Asterisk denotes VSD. *P* value obtained from Fisher’s exact test; scale bar = 200 μm. ND, nondiabetic; DM, diabetic; RA, right atrium; LA, left atrium; RV, right ventricle; LV, left ventricle; IVS, interventricular septum; VSD, ventricular septal defect.

The expression pattern of activated NOTCH1 in the developing heart has been well characterized, being highly expressed within endocardial and endocardially derived mesenchymal cells, which form the endocardial cushions at the AVC and OFT (39, 40). Subsequently, we sought to determine if endothelium-specific *Notch1* haploinsufficiency is sufficient to sensitize the effects of matDM on cardiac development. For this experiment, STZ-induced diabetic and nondiabetic *Notch1^fl/fl^* females were bred with nondiabetic *Tie2-Cre^+^* males to generate control and matDM-exposed *Notch1^fl/wt^ Tie2-Cre^+^* embryos, which are conditionally heterozygous for *Notch1* in all endothelial cells, including endocardial cells and their derived tissues in the heart (Figure 1A). While no VSD was found in nondiabetic *Notch1^fl/wt^ Tie2-Cre^+^* or *Notch1^fl/wt^* littermates, we found a significantly higher incidence of membranous VSD in E14.5 matDM-exposed *Notch1^fl/wt^ Tie2-Cre^+^* embryos (7/17, 41%) compared with non-*Cre^+^* littermates (3/31, 9.7%, Fisher’s exact test *P* value = 0.022) (Figure 1, B and C). To account for variability across multiple matDM-exposed litters (*n* = 6), we used a general linear mixed model and included litter as the random effect, and the probability of VSD for *Notch1^fl/wt^ Tie2-Cre^+^* was found to be higher compared with *Notch1^fl/wt^* (*P* value = 0.06, see Supporting Data Values) (41). While this is slightly higher than the traditional 0.05 cut point, this still small *P* value provides evidence that the data are not compatible with the null hypothesis (42). We also noted that the incidence of VSD in matDM-exposed *Notch1^fl/wt^ Tie2-Cre^+^* (41%) was found to be lower than the incidence observed in matDM-exposed *Notch1^+/–^* (71%). We attribute this difference to potential roles of *Notch1* haploinsufficiency in non-endothelium-derived cells of the heart in the GxE with matDM, or this variability may be due to incomplete deletion of *Notch1* in all endothelial cells. Overall, these results suggest that the observed GxE between *Notch1* haploinsufficiency and matDM can act within the developing endothelium, including the endocardium- and endocardium-derived cells to impair cardiac morphogenesis, particularly ventricular septation.

### Notch1 haploinsufficiency exacerbates the effects of matDM on AV cushion development.

**Figure 2:**
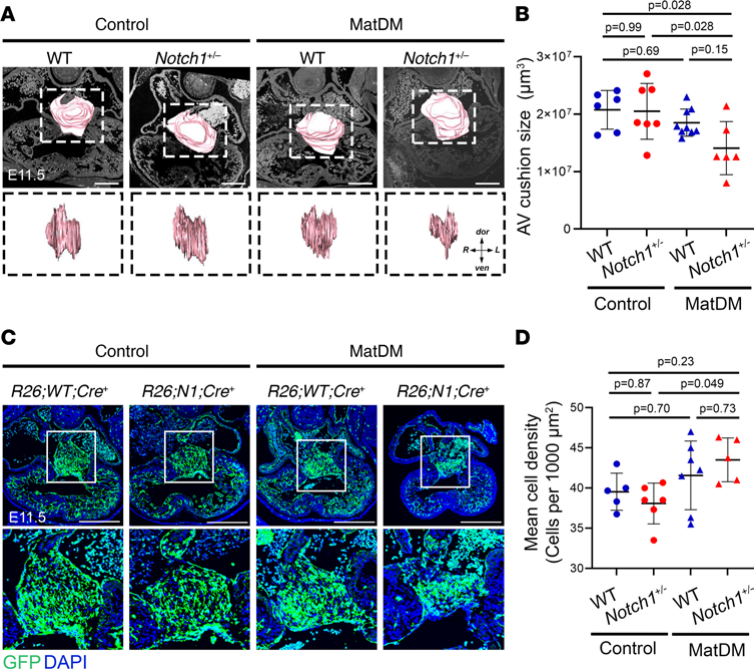
matDM impairs AV cushion size in *Notch1*-haploinsufficient embryonic hearts. (**A**) Three-dimensional (3D) projection of AV cushions in E11.5 control and matDM-exposed WT and *Notch1^+/–^* embryonic hearts. Top panel shows overlay of 3D projection on corresponding 2D transverse section of the heart; scale bar = 100 μm. Bottom panel shows AV cushion 3D projections rotated 90° to show AV cushion span right to left across AVC. (**B**) Quantification of AV cushion volume from 3D projections. (**C**) Control and matDM-exposed WT and *Notch1^+/–^* embryonic hearts showing *Tie2-Cre*–driven *Rosa^mT;mG^*-based GFP expression. GFP^+^ cells in the heart represent all endocardially and endocardially derived cells. Top panel shows GFP expression throughout the embryonic heart; scale bar = 200 μm. Bottom panel shows corresponding 240 × 240 µm zoomed-in images of AV cushions for each genotype. (**D**) Quantification of GFP^+^ cell nuclei within AV cushion based on DAPI staining. Each point represents a single animal of corresponding genotype/condition. *P* values obtained from *t* tests for the estimated marginal means for all pairwise comparisons using Tukey’s correction to adjust for multiple testing. AV, atrioventricular; dor, dorsal; R, right; L, left; ven, ventral.

During cardiac development, endocardial cells undergo EndMT to form endocardial cushions at the AVC and OFT. Subsequent remodeling of the proximal OFT and AV cushions gives rise to the AV septal complex, which includes the mitral and tricuspid valve leaflets and the membranous ventricular septum (24, 25, 43). Here, we sought to determine if interaction between *Notch1* haploinsufficiency and matDM impairs endocardial cushion morphogenesis at the OFT and AVC. As Notch signaling is known to regulate EndMT during endocardial cushion development, we utilized the *Rosa26^mT/mG^* locus to trace endothelial cells that have undergone EndMT in endocardial cushions of matDM-exposed *Notch1^+/–^* embryos compared with nondiabetic controls. We analyzed histological sections of OFT and AV cushions at E11.5; volumetric analysis using 3D reconstruction of serial histological sections revealed matDM-exposed *Notch1^+/–^* hearts had significantly smaller AV cushion size compared with nondiabetic *Notch1^+/–^* and nondiabetic WT hearts (Figure 2, A and B). On the other hand, matDM-exposed WT hearts did not show a statistically significant difference in AV cushion size compared to nondiabetic controls. We found no significant difference in OFT cushion size across the 4 groups, suggesting that this GxE affects AV cushion morphogenesis to contribute to membranous VSD (Supplemental Figure 2, A and B). Upon examination of GFP^+^ EndMT-derived cells within the AV cushions, GFP^+^ cells appeared more compacted within the AV cushion of matDM-exposed *Notch1^+/–^* hearts, which was likely due to the smaller AV cushion size (Figure 2C). This was quantified by counting the number of GFP^+^ cell nuclei within the AV cushion, and a statistically significant increase in cell density was noted between matDM-exposed *Notch1^+/–^* embryos compared with nondiabetic *Notch1^+/–^* control (Figure 2D). Again, this effect was not observed in the matDM-exposed WT littermates, and we noted a high degree of variability in this group, possibly due to the low penetrance of disease in matDM-exposed WT embryos. These results show that the overall size of the AV cushion is significantly smaller in the matDM-exposed *Notch1^+/–^* compared with nondiabetic controls, causing EndMT-derived cells to become more densely packed within the AV cushion.

**Figure 3:**
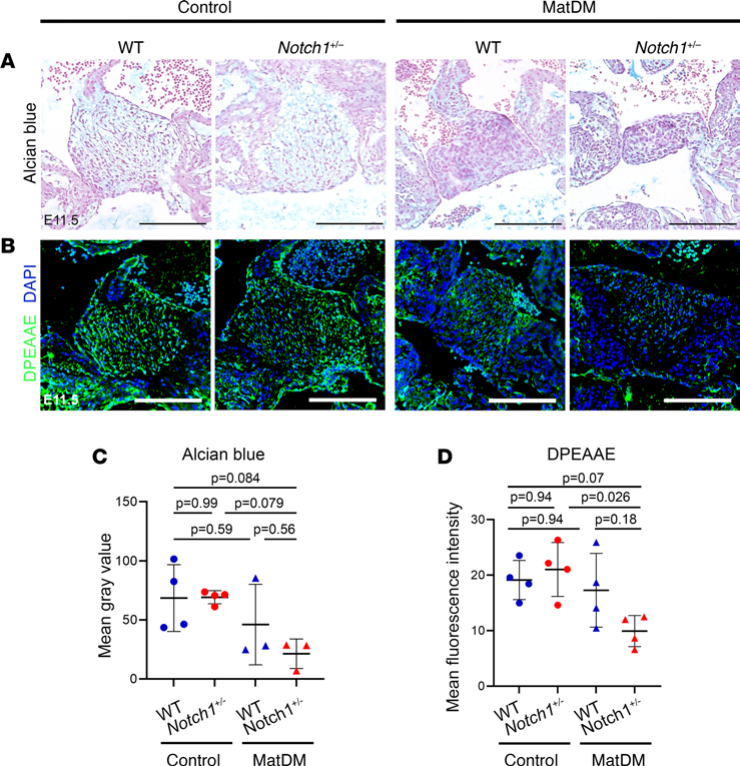
matDM impairs proteoglycan distribution within AV cushion of *Notch1*-haploinsufficient embryonic hearts. (**A**) Alcian blue staining of control and matDM-exposed E11.5 WT and *Notch1^+/–^* AV cushion. (**B**) Immunostaining of cleaved versican (DPEAAE) in control and matDM-exposed WT and *Notch1^+/–^* AV cushion. (**C**) Quantification of Alcian blue staining from **A**. (**D**) Quantification of DPEAAE staining from **B**. Each point represents a single animal of corresponding genotype/condition. *P* values obtained from *t* tests for the estimated marginal means for all pairwise comparisons using Tukey’s correction to adjust for multiple testing. Scale bar = 100 μm.

The deposition and distribution of extracellular matrix (ECM) components, including sulfated proteoglycans such as hyaluronic acid and versican, are crucial for endocardial cushion formation and remodeling into the AV valvuloseptal complex, a process highly regulated by both endocardial and myocardial signaling adjacent to the AV cushion (44–46). Alcian blue staining of WT and *Notch1^+/–^* embryos of diabetic and nondiabetic dams at E11.5 showed reduced proteoglycan deposition in matDM-exposed *Notch1^+/–^* AV cushion compared with nondiabetic control while matDM-exposed WT embryos did not show a difference compared to nondiabetic controls (Figure 3, A and C). During AV cushion development, full-length versican undergoes proteolytic cleavage by the action of matrix metalloproteinases MMP2 and ADAMTS1 (47). Similarly, expression of cleaved versican, labeled by an antibody against neo-epitope DPEAAE, was significantly decreased in the matDM-exposed *Notch1^+/–^* AV cushion compared with nondiabetic *Notch1^+/–^* control (Figure 3, B and D). Similar to the AV cushion size and cellular density data, we found a high degree of variability in the matDM-exposed WT littermates, and as a result no statistical significance was reached between matDM-exposed WT and nondiabetic controls in either the Alcian blue staining or DPEAAE expression. Overall, these results indicate that GxE between *Notch1* haploinsufficiency and matDM impairs AV cushion morphogenesis potentially via disruption of ECM organization.

### NOTCH1 haploinsufficiency acts synergistically with oxidative stress to dysregulate genes involved in EndMT and endocardial cushion morphogenesis.

Previously published research from us and others have proposed matDM-associated oxidative stress as the key driver of cardiac maldevelopment, leading to increased incidence of CHD. To determine if there is elevated oxidative stress in matDM-exposed *Notch1^+/–^* embryos compared with nondiabetic controls, we probed E11.5 matDM-exposed and nondiabetic WT and *Notch1^+/–^* embryonic hearts with an antibody against 4-hydroxynoneal (4-HNE), a stable by-product of lipid peroxidation and a robust biomarker for cellular oxidative stress (48). We detected increased 4-HNE labeling in matDM-exposed *Notch1^+/–^* and WT littermates compared with nondiabetic controls while no difference was detected between *Notch1^+/–^* and WT matDM-exposed hearts, including in the AV cushions, suggesting there are comparable levels of oxidative stress in matDM-exposed WT and *Notch1^+/–^* littermates at this time point (Supplemental Figure 3, A and C). Increased oxidative stress can induce cellular apoptosis in diabetic embryos, particularly during the neurulation stage at E8.75 (49, 50). To probe for increased apoptosis in matDM-exposed WT and *Notch1^+/–^* embryonic hearts, we performed TUNEL assay on E11.5 matDM-exposed and nondiabetic *Notch1^+/–^* and WT embryonic hearts. However, no significant differences were observed across groups, suggesting there is no increase in apoptosis in matDM-exposed cardiac tissues in either *Notch1^+/–^* or WT embryos at this time point (Supplemental Figure 3, B and D).

**Figure 4:**
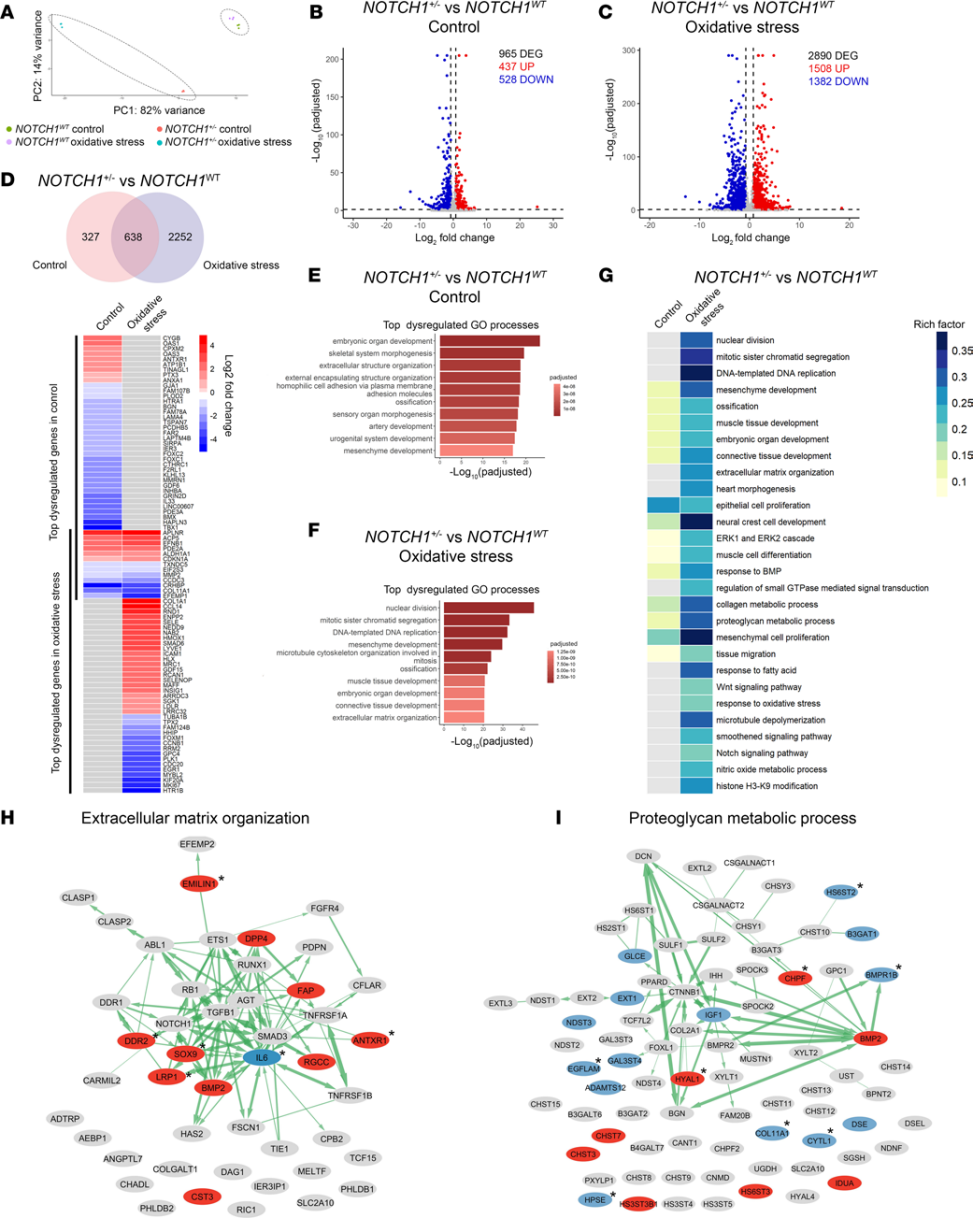
*NOTCH1* haploinsufficiency and oxidative stress act synergistically to dysregulate processes involved in endocardial cushion morphogenesis. (**A**) Principal component analysis shows the grouping of biological replicates and high variance between genotype and response to oxidative stress treatment. (**B**) Volcano plot showing upregulated and downregulated DEGs in *NOTCH1^+/–^* versus *NOTCH1^WT^* iECs in control condition. (**C**) Volcano plot showing upregulated and downregulated DEGs in *NOTCH1^+/–^* and *NOTCH1^WT^* iECs in oxidative stress. (**D**) Venn diagram showing number of common and unique DEGs in *NOTCH1^+/–^* iECs versus *NOTCH1^WT^* in control and oxidative stress condition followed by heatmap showing top 50 significant DEGs in *NOTCH1^+/–^* versus *NOTCH1^WT^* in control and oxidative stress condition. (**E**) Top significantly enriched GO BP terms in *NOTCH1^+/–^* iECs versus *NOTCH1^WT^* in control. (**F**) Top significantly enriched GO BP terms in *NOTCH1^+/–^* iECs versus *NOTCH1^WT^* in oxidative stress. (**G**) Heatmap of dysregulated pathways in *NOTCH1^+/–^* versus *NOTCH1^WT^* in control and oxidative stress. (**H**) DEGs in *NOTCH1^+/–^* iECs versus *NOTCH1^WT^* in oxidative stress that affect GO term extracellular matrix organization. Asterisk indicates genes that were also dysregulated in *NOTCH1^+/–^* iECs versus *NOTCH1^WT^* in control. (**I**) DEGs in *NOTCH1^+/–^* iECs versus *NOTCH1^WT^* in oxidative stress that affect GO term proteoglycan metabolic processes. Asterisk indicates genes that are also significantly differentially expressed in *NOTCH1^+/–^* iECs versus *NOTCH1^WT^* in control. DEGs, differentially expressed genes; FC, fold-change; GO, gene ontology; BP, biological processes; Padj, FDR-adjusted *P* value.

To determine the molecular mechanisms underlying GxE between endothelial *Notch1* and matDM-associated oxidative stress, we utilized *NOTCH1^WT^* and isogenic *NOTCH1^+/–^* iPSC lines, generated as previously described (51, 52). We differentiated *NOTCH1^WT^* and *NOTCH1^+/–^* iPSCs to induced endothelial cells (iECs) using a previously published differentiation protocol (53). Following differentiation, subsets of *NOTCH1^WT^* and *NOTCH1^+/–^* iECs were either exposed to oxidative stress (50 μM H_2_O_2_) or left untreated as control, to mimic matDM-associated oxidative stress in vitro. After 4 days, total RNA was isolated from each iEC subset and bulk RNA sequencing performed for differential gene expression analysis. Principal component analysis of the sequenced samples revealed distinct clustering of biological replicates (*n* = 3 per genotype/condition), with the largest variance observed between *NOTCH1^+/–^* and *NOTCH1^WT^* in oxidative stress (Figure 4A). As expected, we observed high variance between *NOTCH1^+/–^* and *NOTCH1^WT^* control samples, suggesting there are intrinsic transcriptomic differences between *NOTCH1^+/–^* and *NOTCH1^WT^* iECs at baseline untreated condition. Differential gene expression analysis revealed there were 965 genes significantly differentially expressed (FDR-adjusted *P* value < 0.05, absolute log_2_ fold-change > 0.75) between *NOTCH1^+/–^* and *NOTCH1^WT^* iECs in control condition (437 upregulated and 528 downregulated) (Figure 4B and Supplemental Table 1). Next, we turned our attention to how these genotypes differed from each other during exposure to oxidative stress. Differential gene expression analysis revealed there were 2,890 genes significantly differentially expressed (FDR-adjusted *P* value < 0.05, absolute log_2_ fold-change > 0.75) between *NOTCH1^+/–^* and *NOTCH1^WT^* iECs under oxidative stress (1,508 upregulated and 1,382 downregulated) (Figure 4C and Supplemental Table 2). We verified approximately 40% downregulation of *Notch1* mRNA in *NOTCH1^+/–^* iECs under both conditions (log_2_ fold-change = –0.64, FDR-adjusted *P* value < 0.05 in control; log_2_ fold-change = –0.61, FDR-adjusted *P* value < 0.05 in oxidative stress); however, it was not assigned as a DEG because of our stringent log_2_ fold-change cutoff of 0.75. Comparison of DEGs from control and oxidative stress revealed 638 genes were dysregulated in *NOTCH1^+/–^* iECs in both control and oxidative stress while 2,252 genes were dysregulated in *NOTCH1^+/–^* iECs only under oxidative stress (Figure 4D). Overrepresentation analysis of DEGs in *NOTCH1^+/–^* iECs under each condition was performed. Top significant biological processes in *NOTCH1^+/–^* iECs in control condition included embryonic organ development, skeletal system morphogenesis, and extracellular structure organization (Figure 4E and Supplemental Table 3). In contrast, top dysregulated biological processes in *NOTCH1^+/–^* in oxidative stress included terms related to cell division (*MKI67*, *MYBL2*, *CDC20*, *PLK1*, *CCNB1*), mesenchyme development (*APLNR*, *EFNB1*), and extracellular matrix organization (*COL1A1*, *COL11A1*, *MMP2*) (Figure 4F and Supplemental Table 4). While several biological pathways were commonly dysregulated in *NOTCH1^+/–^* iECs in both control and oxidative stress, including GO terms mesenchyme development, connective tissue development, collagen metabolic processes, and proteoglycan metabolic processes, the number of DEGs in each process was higher in *NOTCH1^+/–^* iECs in oxidative stress compared with control condition (Figure 4G). Additionally, many biological processes were found to be dysregulated in *NOTCH1^+/–^* iECs in oxidative stress, including the terms nuclear division, mitotic sister chromatid segregation, extracellular matrix organization, heart morphogenesis, Notch signaling, and nitric oxide signaling pathway among others (Figure 4G). Considering that extracellular matrix organization and proteoglycan metabolic processes are highly relevant processes in the context of endocardial cushion morphogenesis, we investigated the effect of oxidative stress in *NOTCH1^+/–^* iECs on genes within these terms, reporting several interconnected genes to be dysregulated across both processes (Figure 4, H and I). Among DEGs affecting ECM organization, the majority were upregulated, and many of these genes are known to be negative regulators of ECM organization (*DPP4*, *FAP*, *ANTXR1*, *CST3*, *EMILIN1*), while the majority of genes in proteoglycan metabolic processes were downregulated (*BMPR1B*, *IGF1*, *COL11A1*). Taken together, these results suggest *Notch1* haploinsufficiency acts synergistically with oxidative stress to dysregulate gene regulatory networks crucial for endocardial cushion morphogenesis.

### Overexpression of antioxidant gene, SOD1, does not reduce incidence of matDM-associated CHD in the setting of Notch1 haploinsufficiency.

**Figure 5:**
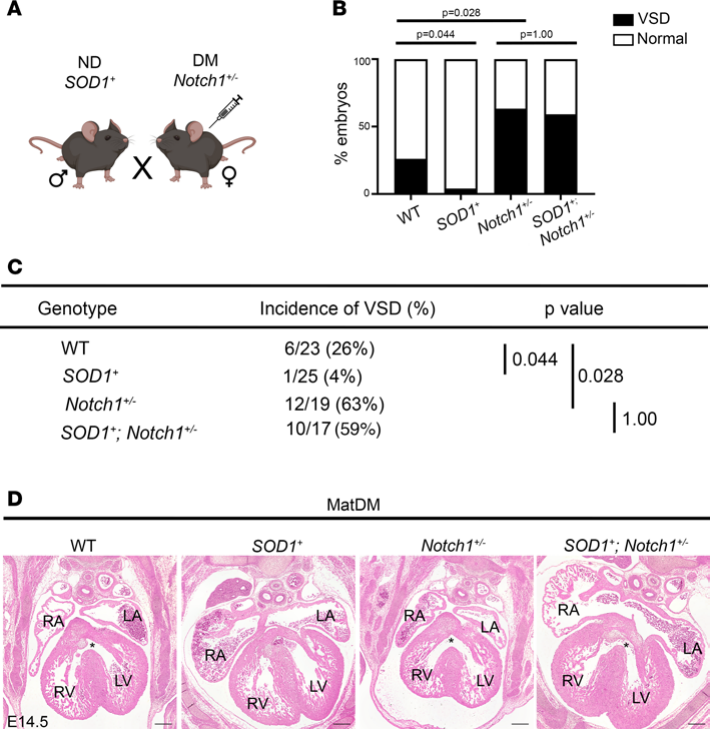
*SOD1* overexpression does not rescue matDM-associated VSD in the setting of *Notch1* haploinsufficiency. (**A**) Breeding scheme to generate E14.5 DM WT, *SOD1^+^*, *Notch1*^+/–^, and *Notch1*^+/–^ *SOD1^+^* embryos. (**B** and **C**) Graph and table showing incidence of VSD in E14.5 embryos by genotype. (**D**) Representative images of matDM-exposed WT, *SOD1^+^*, *Notch1*^+/–^, and *Notch1*^+/–^ *SOD1^+^* embryonic hearts. Asterisk denotes VSD. *P* values obtained from Fisher’s exact test; scale bar = 200 μm. RA, right atrium; LA, left atrium; RV, right ventricle; LV, left ventricle; IVS, interventricular septum; VSD, ventricular septal defect; DM, diabetic.

Oxidative stress is characterized by an imbalance of reactive oxygen species (ROS) production and antioxidant defense response. Superoxide dismutases (SODs) are crucial for neutralizing superoxide radicals, converting them to H_2_O_2_, which can be degraded to H_2_O and O_2_ by endogenous catalase and glutathione peroxidase. To determine if a genetic antioxidant strategy via overexpression of SOD1 is effective in rescuing CHD in a *Notch1^+/–^* background, we bred SOD1-overexpressing transgenic male mice (*SOD1^+^*) to diabetic *Notch1^+/–^* females to generate WT, *Notch1^+/–^*, *SOD1^+^*, and *SOD1^+^ Notch1^+/–^* compound-mutation embryos (*n* = 11 litters) (Figure 5A). We performed histological analysis of E14.5 embryonic hearts and found a significantly decreased incidence of VSD in *SOD1^+^* (1/25) compared with WT (6/23, Fisher’s exact test *P* value = 0.044) (Figure 5, B–D). This is consistent with prior publications showing overexpression of SOD1 in embryonic hearts can reduce the incidence of matDM-associated CHD in a WT setting (31, 32). However, no significant reduction in the incidence of VSD was noted when comparing *Notch1^+/–^* (12/19) and *SOD1^+^ Notch1^+/–^* embryos (10/17, Fisher’s exact test *P* value = 0.99). To account for litter variability, we used binomial regression with litter as a random effect to further analyze these results, and while statistical significance was not achieved, a similar trend was noted (see Supporting Data Values). We compared the level of oxidative stress via 4-HNE labeling between the above genotypes and found decreased immunostaining of 4-HNE in *SOD1^+^* compared with WT while no difference was observed between the *Notch1^+/–^* and *SOD1^+^* *Notch1^+/–^* (Supplemental Figure 4, A and B), suggesting *SOD1* overexpression cannot reduce oxidative stress in a *Notch1^+/–^* background. The failure of *SOD1* overexpression to reduce the incidence of VSD resulting from the *Notch1*-matDM interaction is consistent with our in vitro results showing an exacerbated effect on cardiac developmental process in *NOTCH1^+/–^* iECs exposed to H_2_O_2_. Overall, our results solidify the role of *Notch1* haploinsufficiency as a genetic modifier in matDM-associated CHD, highlighting the presence of GxEs and their mechanisms in CHD pathogenesis.

## Discussion

matDM is an established risk factor for CHD; however, its effect on genetic susceptibilities has not been well described. In this study, we show matDM interacts with *Notch1* haploinsufficiency in vivo to increase the disease penetrance of a membranous VSD phenotype. We find that this GxE acts within the developing endothelial/endocardial and endocardially derived cells and that *Notch1* haploinsufficiency sensitizes the effects of matDM on EndMT and AV cushion morphogenesis, affecting deposition of ECM components including versican. Using a human iPSC-based model, we demonstrate that oxidative stress–exposed *NOTCH1^+/–^* endothelial cells have exacerbated effect on cardiac developmental processes compared with oxidative stress–exposed *NOTCH1^WT^* iECs. We show effects of oxidative stress and *NOTCH1* haploinsufficiency converge to dysregulate networks underlying endocardial cushion morphogenesis. Finally, we find that targeting matDM-associated oxidative stress via overexpression of *SOD1* is unable to rescue VSD in a *Notch1*-haploinsufficient background as compared with successful rescue in WT littermates. Taken together, these results reveal that susceptible genetic variation acts in combination with effects of matDM to disrupt cardiac development and increase the incidence of CHD.

Complex inheritance patterns and incomplete penetrance of CHD are often explained by genetic heterogeneity among individuals, with strong speculation as to the requirement of specific GxE or gene-gene interaction for disease manifestation (6, 54). In humans, pathogenic *NOTCH1* variants are associated with a spectrum of CHD with variable expressivity (33–35). The Notch signaling pathway is highly conserved, and *Notch1* heterozygosity in mice has been reported to interact with both environmental factors (e.g., gestational hypoxia, maternal diabetes) as well as genetic factors (e.g., deletion of *Nos3*) to increase the incidence of heart defects (27, 55, 56). Hence, *NOTCH1* variation may play a crucial role in multifactorial cases of CHD, and results from this study corroborate the role of *NOTCH1* as both a genetic modifier and driver in CHD pathogenesis.

During cardiac development, activated NOTCH1 (N1CD) is expressed in the developing endocardium lining the AVC and OFT; in synchrony with myocardial BMP signaling, N1CD coordinates initiation of EndMT and mesenchyme development at these regions to form the endocardial cushions (37, 57). EndMT-derived mesenchymal cells communicate with adjacent endocardial and myocardial layers to dynamically regulate organization and stratification of the ECM to form the valvuloseptal complex (24, 25). Based on our in vivo results, matDM-exposed *Notch1^+/–^* endocardial cushions do not show any overt signs of initial EndMT failure as lineage tracing does not show a reduction in number of EndMT-derived cells in either matDM-exposed WT or *Notch1^+/–^* littermates compared with nondiabetic controls. Instead, our results suggest that disruption in AV cushion formation results in smaller overall size of the AV cushion in matDM-exposed *Notch1^+/–^* with altered ECM deposition, specifically deposition of cleaved versican. This is consistent with recent reports that have revealed a requirement of N1CD to induce secretion of ECM remodeling and structural proteins within the developing AV cushion (58). Versican is essential for ventricular septum formation, and mice harboring mutant *Versican* exhibit smaller AV cushions and VSD (59). In comparison, matDM-exposed WT littermates displayed great variability in their AV cushion size, cell density, and proteoglycan deposition, with only a subset trending similar to matDM-exposed *Notch1^+/–^* animals, consistent with the low penetrance of disease observed in these animals. In addition, our in vitro findings revealed dysregulation of *BMP2* and *MMP2* expression in *NOTCH1^+/–^* iECs in oxidative stress. While Notch signaling is known to modulate expression of *MMP2*, required for proteolytic cleavage of versican, matDM has also been independently linked to decreased MMP2 expression in the AV cushions (28, 60). Endocardial BMP2 has also been shown to be essential for later AV cushion morphogenesis, regulating deposition of ECM components such as *Col9a1*, *Has2*, *Postn*, and *Vcan* (61). Our results suggest *Notch1* haploinsufficiency can sensitize the genetic network underlying ECM organization during AV cushion development with exposure to matDM-associated oxidative stress.

Although diabetes mellitus is characterized by chronic hyperglycemia, diabetic embryopathy is likely driven by a combination of metabolic imbalances occurring within the intrauterine diabetic milieu. Diabetes mellitus is associated with disruption of several metabolic pathways, including glycolysis, sorbitol production, ketone formation, and increased synthesis of advanced glycation end-products, all of which result in increased ROS production and cellular oxidative stress (62–64). As expected, matDM-exposed *Notch1^+/–^* embryos exhibit oxidative stress comparable to matDM-exposed WT littermates, as shown by 4-HNE labeling. Antioxidant therapy has been tested by several research groups, including us, in an attempt to decrease the incidence of matDM-associated CHD, but lack of consensus on route of administration and/or strategy (genetic versus pharmacologic) has led to inconsistent results (27, 32, 65). In this study, we find *SOD1* overexpression can only decrease the incidence of VSD in a WT background and cannot rescue VSD in a *Notch1*-haploinsufficient setting. Based on 4-HNE labeling across matDM-exposed littermates, this seems to be due to inability of SOD1 to effectively reduce ROS levels below the required threshold for rescue in matDM-exposed *Notch1^+/–^* mice. Based on our in vitro studies, processes such as cell division and apoptosis were among the top significantly affected pathways in *NOTCH1*-haploinsufficient iECs exposed in H_2_O_2_ along with ECM organization. Proliferation and apoptosis of mesenchymal cells within the endocardial cushions is carefully coordinated with the start of ECM stratification (66). Previous studies have shown increased apoptosis and decreased proliferation in embryonic hearts exposed to maternal diabetes, particularly at later stages of endocardial cushion development. However, we did not find increased TUNEL^+^ apoptotic cells in matDM-exposed WT and *Notch1^+/–^* compared to nondiabetic controls, possibly due to small sample size and timing during embryonic development. Our in vitro results show decreased expression of *BMP4* specifically in *NOTCH1^+/–^* iECs exposed to H_2_O_2_, and BMP4 has been reported to mediate regulation of endocardial cushion apoptosis (30, 67). As such, we speculate that a combination of dysregulated ROS/Notch signaling contributes to the magnified effect on cell division and cell death in *NOTCH1* haploinsufficiency and may be acting synergistically to cause VSD in vivo, thus making it unable to be rescued by *SOD1* overexpression alone.

Results from the in vitro experiments demonstrated relevant transcriptomic changes related to endocardial cushion morphogenesis in *NOTCH1^+/–^* compared with *NOTCH1^WT^* iECs in the control condition, although they were not as affected as found in oxidative stress conditions. This supports the notion that a genetic hit in *NOTCH1* can “prime” the cell to a secondary environmental hit. Although *Notch1^+/–^* mice are not reported to have a congenital cardiac phenotype, they are known to have impaired lipid metabolism and develop aortic aneurysms with age (68, 69). The lack of CHD in nondiabetic *Notch1^+/–^* mice may be explained by compensatory mechanisms occurring in vivo that cannot be recapitulated in our in vitro endothelial cell-autonomous system.

In summary, our work uncovers a composite effect of genetic variation and exposure to an environmental teratogen on cardiac morphogenesis. We report that these effects can disrupt specific cardiac developmental networks in a cell lineage–specific manner. Furthermore, we have shown that broad therapeutic strategies, such as the use of antioxidants, may not be effective in genetically sensitized populations. Future studies should aim to uncover other potential GxEs that may be contributing to matDM-associated CHD. Studies of this nature will pave the way for personalized therapy to improve outcomes from diabetic pregnancies in genetically susceptible individuals and reduce the risk of matDM-associated CHD.

This study has limitations. In this study, we defined diabetic mice as those having fasting blood glucose greater than 200 mg/dL. However, we were not able to monitor or control blood glucose levels throughout pregnancy and attribute a degree of variability in the matDM-exposed samples due to differences in maternal glucose levels. We have performed binomial regression analysis with litter as a random factor to increase confidence in our results. In this study, we used C57BL/6J mice for all in vivo studies. However, strain-related differences may lead to variable phenotypic results. In the context of GxE, it is important to determine whether *Notch1* haploinsufficiency in a different strain/genetic background can also interact with matDM to increase CHD incidence. Consequently, we focused on the effect of GxE in AV cushion development as our phenotype was limited to membranous VSDs, and we found no significant difference in OFT cushions. Future studies with other animal strains may identify additional cardiac phenotypes that may require investigation into other cardiac developmental processes. Additionally, we focused on endothelial/endocardial and endocardially derived cell–specific GxE as *Notch1* expression is enriched in this cell lineage. However, NOTCH1 can also play a role during cardiomyocyte differentiation and specification, and investigation of the nonendothelial roles of *Notch1* in the context of matDM is beyond the scope of this paper and will be reserved for future investigations. For our in vitro studies, all experiments were conducted using iPSC lines derived from a single healthy donor. Whether similar results will be observed with multiple donor cell lines is yet to be determined.

## Methods

### Sex as a biological variable.

Our study did not consider sex to be a biological variable as our studies involved evaluation of embryonic hearts.

### Experimental mouse models.

WT C57BL/6 (Jackson Laboratory [Jax], 000664), *Rosa26^mTmG^* (Jax, 007676), *Tie2-Cre^+^* (Jax, 004128), and *SOD1-tg* (Jax, 002297) animals were purchased. *Notch1*^+/–^ and *Notch1^fl/fl^* mice were generated and genotyped as described previously (70, 71). *Rosa^+/+^ Notch1^+/–^* compound mutants were generated by intercrossing *Rosa26^mTmG^* with *Notch1^+/–^* mice. Six- to 8-week-old female *WT*, *Notch1^fl/fl^*, *Rosa^+/+^ Notch1^+/–^*, and *Notch1^+/–^* mice were used to induce diabetes mellitus by intraperitoneal injection of STZ (NC0146241, Thermo Fisher Scientific) dissolved in 0.01 mol/L citrate buffer, pH 4.5, at 75 mg/kg body weight for 3 consecutive days. Two weeks after STZ injection, mice were fasted for 8 hours during the light cycle, and glucose levels were measured using the AlphaTrak 2 veterinary blood glucometer calibrated specifically for rodents from tail vein blood (Abbott Laboratories). Mice with fasting blood glucose greater than 200 mg/dL (11 mmol/L) were defined as diabetic and used for timed breeding and embryo collections.

### Mouse embryo collection.

Timed mating was performed using the following pairs: nondiabetic *Notch1^+/–^* males and diabetic WT females, nondiabetic *Tie2-Cre^+^* males with diabetic and nondiabetic *Notch1^fl/fl^* females or diabetic and nondiabetic *Rosa^+/+^ Notch1^+/–^* females, and nondiabetic *SOD1-tg* males with diabetic *Notch1^+/–^* females. Mice were maintained on a 12-hour-light/12-hour dark cycle, with noon of the day of vaginal plug observation defined as E0.5. Pregnant dams were euthanized using isoflurane, and embryos were collected at E11.5 and E14.5.

### Embryo processing and histological analysis.

Harvested embryos were fixed in 4% paraformaldehyde at 4°C overnight followed by alcohol gradient dehydration and paraffin embedding. Serial tissue sections (6 μm) of the embryonic heart were collected for histological analysis. H&E staining (Vector Laboratories) was used to visualize gross morphology and Alcian blue staining (Vector Laboratories) to visualize proteoglycan deposition. Images were taken using Keyence BZ-X810 Fluorescence Microscope.

### Amira3D reconstruction of developing endocardial cushions and cell counts.

Serial sections of embryonic hearts from diabetic and nondiabetic group were imaged at 10× original magnification using Olympus BX51, and images were loaded onto Amira3D v2023.2 for alignment and segmentation of AV endocardial cushions. Volume measurement was recorded using the MaterialStatistics module. To count EndMT-derived cells within the AV cushion, diabetic and nondiabetic E11.5 *Rosa^mTmG^; WT; Tie2-Cre^+^* and *Rosa^mTmG^; Notch1^+/–^; Tie2-Cre^+^* (Figure 2C) sections were used. Following immunostaining against GFP, the number of cells in the AV cushion was quantified by counting nuclei stained with DAPI of GFP^+^ cells within the defined area of the AV cushion. At least 3 readings were taken from each image, and 3 serial sections were used for each biological replicate to obtain mean cell count/area within the AV cushion.

### Immunofluorescence and immunohistochemistry.

Histological sections of embryonic hearts were deparaffinized using xylene and gradient ethanol washes, followed by antigen retrieval using heat-mediated, citrate-based Antigen Unmasking solution (H-3300, Vector Laboratories). For immunofluorescence, tissue sections were blocked with 2% normal horse serum in 1× phosphate-buffered saline with 0.1% Tween 20 (PBST) for 1 hour and then incubated with rabbit anti-GFP (Abcam ab290, 1:200) and rabbit anti-DPEAAE (Invitrogen PA1-1748A, 1:100) overnight at 4°C. Following 1× PBST wash, sections were incubated with donkey anti-rabbit secondary antibody conjugated to Alexa Fluor 488 for 1 hour at room temperature in the dark (Invitrogen A-21206). Sections were washed 3 times with 1× PBST and counterstained with VECTASHIELD Vibrance Mounting Medium with DAPI (Vector Laboratories). Fluorescence images were visualized using the Keyence BZX-810 Fluorescence Microscope. Relative fluorescence intensity was measured using ImageJ software (NIH). For immunohistochemistry, unmasked tissue sections were incubated in 3% H_2_O_2_ followed by blocking in 1% bovine serum albumin in 1× PBST for 1 hour and then incubated with mouse anti–4-HNE (R&D Systems, Bio-Techne; MAB3249, 1:100) overnight at 4°C. Following 1× PBST wash, sections were incubated with SignalStain Boost IHC detection reagent (HRP anti-mouse; Cell Signaling Technology 8125) for 1 hour at room temperature. SignalStain DAB substrate kit (Cell Signaling Technology 8059) was used to develop the stain. Images were taken using the Keyence BZX-810 Microscope in bright-field. Mean gray value of each image was measured using ImageJ software.

### Cellular apoptosis.

TUNEL assay was performed to detect cellular apoptosis using TUNEL Assay Kit (Cell Signaling Technology 64936S) according to manufacturer’s instructions. For nuclear staining, slides were mounted using VECTASHIELD Vibrance Mounting Medium with DAPI. Fluorescence images were taken using Keyence BZ-X810 Fluorescence Microscope, and percentage of TUNEL^+^ cells within the embryonic heart was counted using ImageJ software.

### Culture of iPSCs, directed differentiation into endothelial lineage, and oxidative stress treatments.

*NOTCH1^WT^* and *NOTCH1^+/–^* iPSCs were generated as previously described (51, 52). For differentiation into endothelial cells, *NOTCH1^WT^* and *NOTCH1^+/–^* iPSCs were cultured in complete E8 media until approximately 90% confluent and then changed to differentiation media following a published protocol (53). For oxidative stress studies, H_2_O_2_ was added to EGM2 media at a final concentration of 50 μM, and cells were exposed for 4 days, with media refreshment every 24 hours.

### RNA-Seq analysis.

Following oxidative stress treatment for 4 days, total RNA was collected from *NOTCH1^WT^* and *NOTCH1^+/–^* iECs using Total RNA Purification Kit (Norgen Biotek) according to manufacturer’s instructions. RNA was prepared for sequencing following provider’s instructions (Novogene Corporation) and sequenced on a NovaSeq 6000 (Illumina). Primary processing and expression quantification of the reads were performed by DataPrudence (Carboctet). FASTQ files were trimmed to remove Illumina adaptor sequences and low-quality reads using TrimGalore! in “paired” mode (72). Paired, trimmed reads were then aligned against *Homo sapiens* assembly version GRCh38.p14, GENCODE 44 (http://Jul2023.archive.ensembl.org/Homo_sapiens/Info/Annotation), using the STAR alignment tool (v2.7.10b) with “--quantMode TranscriptomeSAM” option (73). The transcriptome-aligned BAM was used to collect gene-level and transcriptome-level count estimates using RSEM (v1.3.1) (74). The gene count estimates were used to identify DEGs using DESeq2 (v1.34.0) (75). Overrepresentation analysis was performed using R package genekitr (https://CRAN.R-project.org/package=genekitr) and cross-validated with clusterProfiler (76). GO terms were simplified using GO semantic similarity analysis using built-in GOsim() function in genekitr (77). Volcano plots were created using ggplot2 (78). Gene regulatory networks were visualized and annotated using Cytoscape (79).

### Statistics.

Categorical data are presented with count and percentage while continuous data are presented as mean and SD. Our experiments included embryos from multiple diabetic dams, which introduces the possibility that correlation of outcomes from embryos from the same litter could impact results. For binomial outcomes (incidence of VSD in Figures 1 and 5), Fisher’s exact test was used, and binomial regression models with robust standard errors accounting for litter were conducted as sensitivity analyses (Supporting Data Values). For continuous outcomes, linear regression with random effects for litter were conducted, and 2-tailed *t* tests for the estimated marginal means for all pairwise comparisons were carried out using Tukey’s correction to adjust for multiple testing. A *P* value less than 0.05 was considered statistically significant.

### Study approval.

All animal use was approved and monitored on protocol AR09-00056 by the Institutional Animal Care and Use Committee at the Research Institute at Nationwide Children’s Hospital and conducted in accordance with the NIH’s *Guide for the Care and Use of Laboratory Animals* (National Academies Press, 2011).

### Data availability.

Values for all data points shown in graphs are reported in the Supporting Data Values file. The RNA-Seq data produced in this study were deposited to the NCBI’s Gene Expression Omnibus database (accession no. GSE279323).

## Author contributions

VG and TZC conceived the study and experimental design with input from DL and MTZ. TZC and SCG performed and analyzed the experiments. The murine studies were performed by TZC, SCG, HBG, and EMC. MTZ generated and provided iPSC lines. Bioinformatics analyses were performed by TZC, ASR, and YB. Statistical analysis was performed by TZC, SC, and VG. TZC and VG wrote the manuscript with input from all authors.

## Supplementary Material

Supplemental data

Supplemental table 1

Supplemental table 2

Supplemental table 3

Supplemental table 4

Supporting data values

## Version 1 10/22/2024

In-Press Preview

## Version 2 12/06/2024

Electronic publication
