# Supplementary material for: Impact of genetic factors on antioxidant rescue of maternal diabetes–associated congenital heart disease
Source: JCI Insight. 2024 Dec 6;9(23):e183516. doi: 10.1172/jci.insight.183516 (PMC11623948; doi:10.1172/jci.insight.183516)
Supplement: Supplemental data [file jciinsight-9-183516-s136.pdf]

Supplemental Materials

Title: Impact of genetic factors on antioxidant rescue of maternal diabetes-associated congenital heart disease

Authors: Talita Z. Choudhury[1,2], Sarah C. Greskovich[1], Holly B. Girard[1], Anupama S. Rao[1], Yogesh Budhathoki[2], Emily M. Cameron[1], Sara Conroy[3,4], Deqiang Li[1,4], Ming-Tao Zhao[1,4], Vidu Garg[1,4,5]

Supplemental Figure Legends

Supplemental Figure 1. Gene-environment interaction between maternal diabetes mellitus and *Notch1* haploinsufficiency increases penetrance of ventricular septal defects. (A) Breeding scheme to generate E14.5 WT and *Notch1*$^{+/-}$ embryos for histological analysis. (B) Table showing incidence of VSD in E14.5 embryos. (C) Representative images of matDM-exposed WT and *Notch1*$^{+/-}$ embryonic hearts. Asterisk denotes VSD. ND, Non-diabetic; DM, diabetic; RA, right atrium; LA, left atrium; RV, right ventricle; LV, left ventricle; IVS, interventricular septum; VSD, ventricular septal defect. *p*-values obtained from Fisher's exact test; scale bar = 200 µm.

Supplemental Figure 2. Gene-environment interaction between maternal diabetes mellitus and *Notch1* haploinsufficiency does not affect endocardial cushions of the outflow tract. (A) Three-dimensional (3D) projection of OFT cushions in E11.5 control and matDM-exposed WT and *Notch1*$^{+/-}$ embryonic hearts. Top panel shows overlay of 3D

projection on corresponding 2D transverse section of the heart; scale bar = 100 µm. (B) Quantification of OFT cushion volume from 3D projections. Each point represents a single animal of corresponding genotype/condition. P values obtained from t-tests for the estimated marginal means for all pairwise comparisons using Tukey's correction to adjust for multiple testing; OFT, outflow tract.

Supplemental Figure 3. Oxidative stress and cellular apoptosis in WT and *Notch1*$^{+/-}$ embryonic hearts exposed to maternal diabetes mellitus. (A) Immunostaining against 4-HNE in control and matDM-exposed WT and *Notch1*$^{+/-}$ at E11.5. (B) TUNEL assay of control and matDM-exposed WT and *Notch1*$^{+/-}$ at E11.5. White arrows indicate TUNEL+ cells (C) Quantification of (A). (D) Quantification of (B). Each dot represents single animal of corresponding genotype/condition. WT, wildtype; ND, non-diabetic; DM, diabetic; p values obtained from t-tests for the estimated marginal means for all pairwise comparisons using Tukey's correction to adjust for multiple testing.

Supplemental Figure 4. Oxidative stress can be modulated by SOD1 overexpression. (A) Immunostaining of 4-HNE in E14.5 matDM-exposed WT*, SOD1*$^{+}$*, Notch1*$^{+/-}$*, Notch1*$^{+/-}$*;SOD1*$^{+}$ embryonic hearts (B) Quantification of (A). Each dot represents single animal of corresponding genotype. WT, wildtype; DM, exposed to maternal diabetes; p values obtained from t-tests for the estimated marginal means for all pairwise comparisons using Tukey's correction to adjust for multiple testing. Scale bar = 200 µm.

Supplemental Tables:

Extended Table 1 - Differentially expressed genes in *NOTCH1*$^{+/-}$ vs *NOTCH1*^{WT} in control condition

Extended Table 2 - Significantly dysregulated biological processes in *NOTCH1*$^{+/-}$ vs *NOTCH1*^{WT} in control condition

Extended Table 3 - Differentially expressed genes in *NOTCH1*$^{+/-}$ vs *NOTCH1*^{WT} in oxidative stress

Extended Table 4 - Significantly dysregulated biological processes in *NOTCH1*$^{+/-}$ vs *NOTCH1*^{WT} in oxidative stress

Supporting Data Values

Supplemental Figures

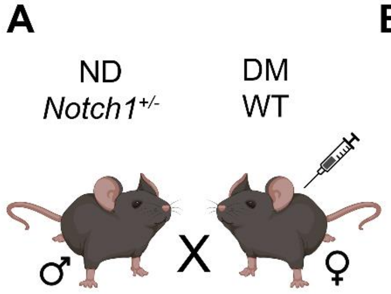

B

Genotype	Incidence of VSD (%)	p value
WT	1/10 (10%)	0.005
Notch1$^{+/-}$	10/14 (71%)	

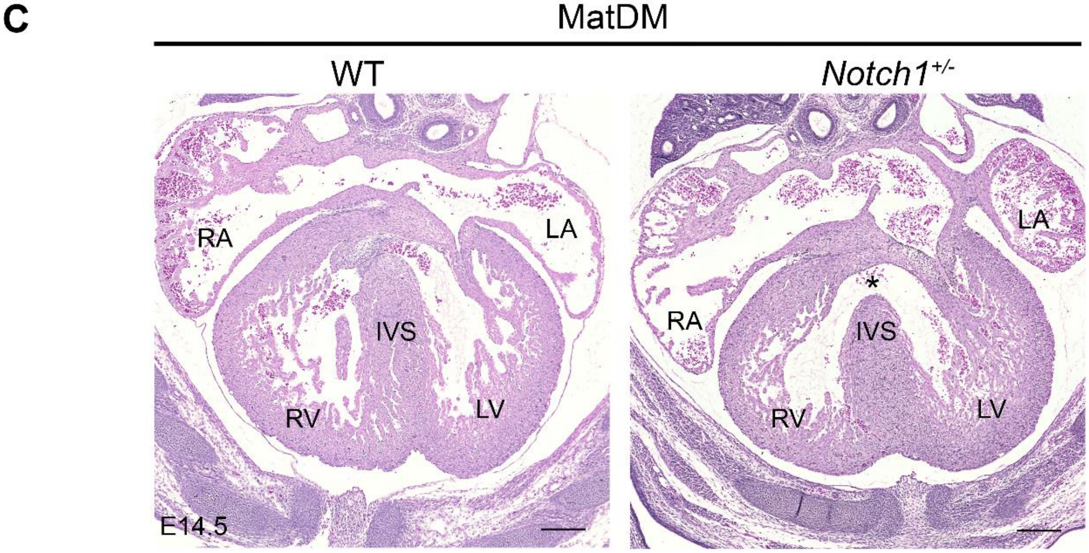

Supplemental Figure 1. Gene-environment interaction between maternal diabetes mellitus and *Notch1* haploinsufficiency increases penetrance of ventricular septal defects. (A) Breeding scheme to generate E14.5 WT and *Notch1*$^{+/-}$ embryos for histological analysis. (B) Table showing incidence of VSD in E14.5 embryos. (C) Representative images of matDM-exposed WT and *Notch1*$^{+/-}$ embryonic hearts. Asterisk denotes VSD. ND, Non-diabetic; DM, diabetic; RA, right atrium; LA, left atrium; RV, right ventricle; LV, left ventricle; IVS, interventricular septum; VSD, ventricular septal defect. P values obtained from Fisher's exact test; scale bar = 200 µm.

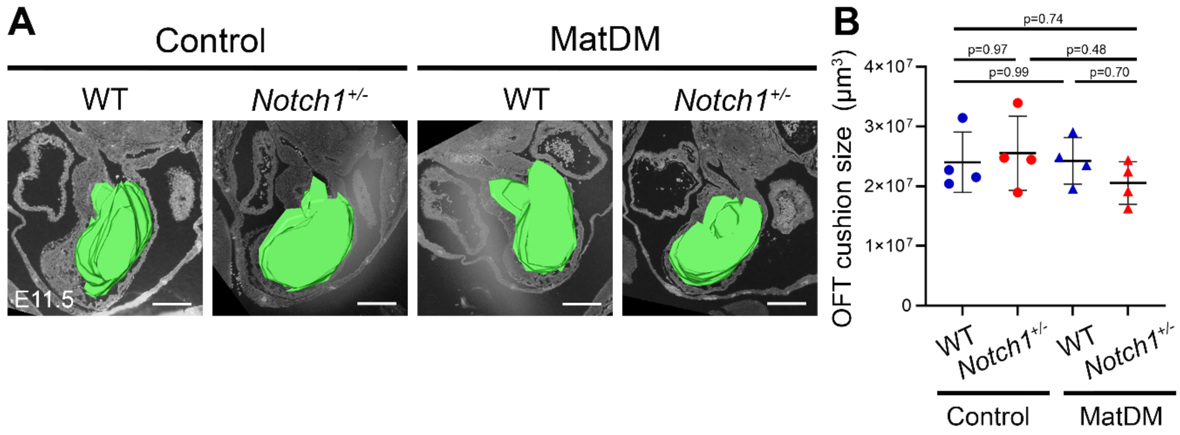

Supplemental Figure 2. Gene-environment interaction between maternal diabetes mellitus and *Notch1* haploinsufficiency does not affect endocardial cushions of the outflow tract. (A) Three-dimensional (3D) projection of OFT cushions in E11.5 control and matDM-exposed WT and *Notch1*$^{+/-}$ embryonic hearts. Top panel shows overlay of 3D projection on corresponding 2D transverse section of the heart; scale bar = 100 µm. (B) Quantification of OFT cushion volume from 3D projections. Each point represents a single animal of corresponding genotype/condition. P values obtained from t-tests for the estimated marginal means for all pairwise comparisons using Tukey's correction to adjust for multiple testing; OFT, outflow tract.

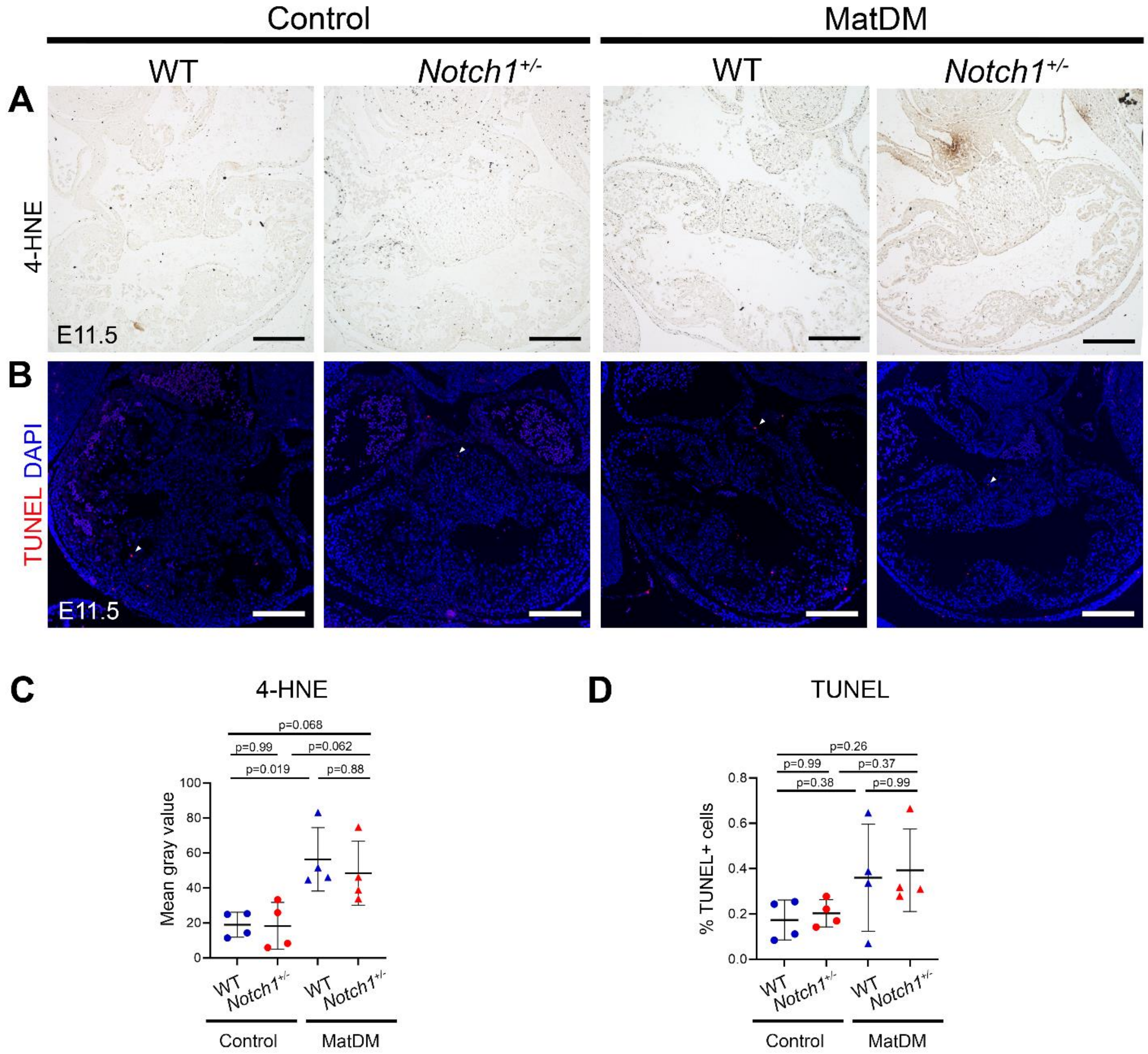

Supplemental Figure 3. Oxidative stress and cellular apoptosis in WT and *Notch1*$^{+/-}$ embryonic hearts exposed to maternal diabetes mellitus. (A) Immunostaining against 4-HNE in control and matDM-exposed WT and *Notch1*$^{+/-}$ at E11.5. (B) TUNEL assay of control and matDM-exposed WT and *Notch1*$^{+/-}$ at E11.5. White arrows indicate TUNEL+ cells (C) Quantification of (A). (D) Quantification of (B). Each dot represents single animal of corresponding genotype/condition. WT, wildtype; ND, non-diabetic; DM, diabetic; p values obtained from t-tests for the estimated marginal means for all pairwise comparisons using Tukey's correction to adjust for multiple testing.

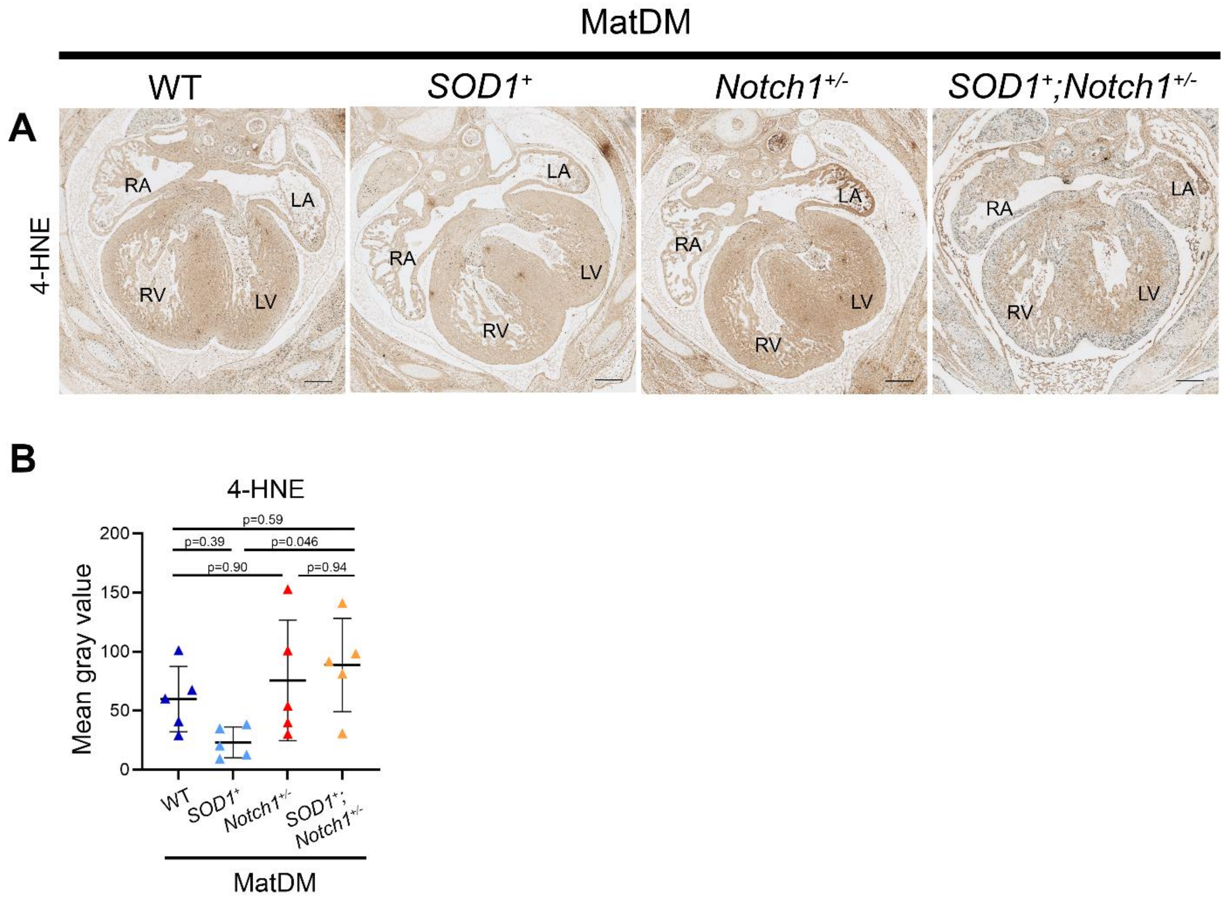

Supplemental Figure 4. Oxidative stress can be modulated by SOD1 overexpression. (A) Immunostaining of 4-HNE in E14.5 matDM-exposed WT, *SOD1⁺*, *Notch1⁺/⁻*, *Notch1⁺/⁻;SOD1⁺* embryonic hearts (B) Quantification of (A). Each dot represents single animal of corresponding genotype. WT, wildtype; DM, exposed to maternal diabetes; p values obtained from t-tests for the estimated marginal means for all pairwise comparisons using Tukey's correction to adjust for multiple testing. Scale bar = 200 µm.
